# Seroprevalence of Human Betaretrovirus Surface Protein Antibodies in Patients with Breast Cancer and Liver Disease

**DOI:** 10.1155/2020/8958192

**Published:** 2020-01-27

**Authors:** Guangzhi Zhang, Kiandokht Bashiri, Mark Kneteman, Kevan Cave, Youngkee Hong, John R. Mackey, Harvey J. Alter, Andrew L. Mason

**Affiliations:** ^1^Center of Excellence for Gastrointestinal Inflammation and Immunity Research, Division of Gastroenterology, University of Alberta, Edmonton, AB T6G 2E1, Canada; ^2^National Microbiology Laboratory, Winnipeg, MB R3E 3M4, Canada; ^3^Department of Medical Oncology, Cross Cancer Institute, University of Alberta, Edmonton, AB, Canada; ^4^Department of Transfusion Medicine, National Institutes of Health, Bethesda, MD 20892, USA; ^5^Li Ka Shing Institute of Virology, University of Alberta, Edmonton, AB T6G 2E1, Canada

## Abstract

Mouse mammary tumor virus (MMTV) is a betaretrovirus that plays a causal role in the development of breast cancer and lymphoma in mice. Closely related sequences that share 91–99% nucleotide identity with MMTV have been repeatedly found in humans with neoplastic and inflammatory diseases. Evidence for infection with a betaretrovirus has been found in patients with breast cancer and primary biliary cholangitis and referred to as the human mammary tumor virus and the human betaretrovirus (HBRV), respectively. Using the gold standard technique of demonstrating retroviral infection, HBRV proviral integrations have been detected in cholangiocytes, lymph nodes, and liver of patients with primary biliary cholangitis. However, the scientific biomedical community has not embraced the hypothesis that MMTV like betaretroviruses may infect humans because reports of viral detection have been inconsistent and robust diagnostic assays are lacking. Specifically, prior serological assays using MMTV proteins have produced divergent results in human disease. Accordingly, a partial HBRV surface (Su) construct was transfected into HEK293 to create an ELISA. The secreted HBRV gp52 Su protein was then used to screen for serological responses in patients with breast cancer and liver disease. A greater proportion of breast cancer patients (*n* = 98) were found to have serological reactivity to HBRV Su as compared to age- and sex-matched control subjects (10.2% versus 2.0%, *P*=0.017, OR = 5.6 [1.25–26.3]). Similarly, the frequency of HBRV Su reactivity was higher in patients with primary biliary cholangitis (*n*  =  156) as compared to blood donors (11.5% vs. 3.1%, *P*=0.0024, OR = 4.09 [1.66–10.1]). While the sensitivity of the HBRV Su ELISA was limited, the assay was highly specific for serologic detection in patients with breast cancer or primary biliary cholangitis, respectively (98.0% [93.1%–99.7%] and 97.0% [93.4%–98.6%]). Additional assays will be required to link immune response to betaretrovirus infection and either breast cancer or primary biliary cholangitis.

## 1. Introduction

Breast cancer is the most frequent cancer diagnosis among females and a leading cause of cancer deaths worldwide [1, 2]. Several viruses have been linked with the development of human breast cancer, but none have been established as having a causal etiology [3, 4]. One such agent resembles mouse mammary tumor virus (MMTV), a murine betaretrovirus that plays a direct role in the development of breast cancer in mice [5]. Indeed, cloned betaretrovirus nucleotide sequences from humans reportedly share between 91% and 99% identity with various regions of the MMTV genome [6–9]. However, diagnostic assays are lacking to reproducibly detect betaretrovirus infection in humans [10].

MMTV does not encode an oncogene but rather activates growth pathways by insertional mutagenesis to promote carcinogenesis in mice [11]. The diagnosis of MMTV infection in mice can be challenging. The viral burden is below the limits of detection in blood, and the agent is encoded as an endogenous retrovirus in most mice; therefore, exogenous viral genomic nucleic acid sequences cannot easily be distinguished from the endogenous expression of MMTV [12]. Furthermore, inadequate humoral responses are made by weanling pups infected via ingestion of MMTV in milk due to the tolerizing effects of neonatal infection by the oral route [13]. Accordingly, a diagnosis of MMTV infection is made by assessing the skewing of T-cell receptor V-*β* subsets to demonstrate the MMTV superantigen effect [14].

Evidence for human infection first surfaced in 1971, when B-type particles resembling MMTV were observed by electron microscopy in the milk of breast cancer patients [15]. Breast cancer patients were also reported to harbor betaretrovirus nucleic acid sequences and/or proteins in various samples, including milk [16], serum [17], salivary glands [18], as well as breast cancer tissue [19], cyst fluid [20], and breast cancer cells in culture [21, 22]. Thereafter, betaretrovirus sequences resembling MMTV were PCR-cloned from breast cancer tissues derived from various countries, and the agent was referred to as the human mammary tumor virus [7, 23–27].

In 2003, a human betaretrovirus (HBRV) was characterized in patients with primary biliary cholangitis (PBC; previously known as primary biliary cirrhosis [28]), an inflammatory autoimmune liver disease. The agent was predominantly detected in perihepatic lymph nodes and was shown to promote the expression of mitochondrial autoantigens in cocultivation studies with cholangiocytes, a well-characterized PBC disease-specific phenotype [9, 29]. Evidence of human betaretrovirus proviral integrations was subsequently demonstrated in PBC patients by ligation-mediated PCR and Illumina sequencing, using a bioinformatics pipeline that ensured the exclusion of all sequences potentially related to murine or HERV sequences. More than 2,200 unique HBRV integrations were identified, and the majority of PBC patients were found to have evidence of proviral integrations linked with HBRV RNA production in cholangiocytes [30]. In clinical trials, PBC patients on combination antiretroviral therapy have shown biochemical and histological improvement with therapy [31–34].

The hypothesis that a betaretrovirus may be linked with human breast cancer has gained little traction over the years because of the inconsistency of findings in different reports, a concern for cross-reactivity with human endogenous retroviruses (HERV) and the low level of viral burden [10, 35, 36]. With regard to the potential for a link with betaretrovirus infection and PBC, investigators have either been unable to detect viral infection [37] or to confirm the specificity of HBRV infection in PBC patients [38]. Furthermore, serological studies using MMTV preparations as substrate have been unable to demonstrate specific antibody reactivity to defined MMTV proteins [37, 39]. While HBRV shares between 93% and 97% amino acid identity with the MMTV envelope protein, consistent differences have been observed between HBRV Env compared to MMTV Env that may alter antigenicity [6]. In the present study, we expressed the HBRV gp52 surface (Su) protein in human cells to create an enzyme-linked immunosorbent assay (ELISA). Herein, we report the seroprevalence of anti-HBRV gp52 Su reactivity in patients with breast cancer, patients with liver disease, and healthy subjects.

## 2. Materials and Methods

### 2.1. Ethics

The study protocol was approved by the Human Ethics Review Board from the University of Alberta and institutional review boards/ethics committees at each site. The project was conducted in accordance with the Declaration of Helsinki (1964).

### 2.2. Patient Samples

A serum panel of breast cancer patients (*n* = 98) and age/sex-matched controls (*n* = 102) was obtained from the Alberta Tomorrow Project, a longitudinal study tracking 55,000 adults in Alberta [40]. Liver disease patient serum was prospectively collected from the hepatology outpatients at the Zeidler Clinic, University of Alberta Hospital from January 2003 to December 2014. Serum from 156 patients with PBC, 46 with primary sclerosing cholangitis (PSC), 16 with autoimmune hepatitis (AIH), 25 with nonalcoholic fatty liver disease (steatosis), 8 with alcoholic liver disease (ALD), 19 with viral hepatitis, 6 with cryptogenic liver disease, and 19 with miscellaneous liver disease. Healthy blood donors' serum samples (*n* = 194) were provided by the Department of Transfusion Medicine, National Institute of Health, Bethesda, MD.

### 2.3. Recombinant DNA Expression Constructs

The HBRV Su was derived from HBRV sequences obtained from a PBC patients' perihepatic lymph node [6]. The HBRV Su coding sequence was cloned into pcDNA3.1 (Invitrogen) vector along with a TAP tag at the 3′ terminus of the HBRV Su [41] and 4 copies of M-PMV cytoplasmic transport element (CTE) downstream [42]. The expressed HBRV envelope protein sequence corresponds to amino acids 99 to 455 in the surface region that includes the receptor-binding domain, which shares 97% and 98% amino acid identity with MMTV Su [6] (see supplementary material for HBRV Su and MMTV Su alignment; Supplementary Figure 1).

### 2.4. Cell Culture, Transfection, and Stable Cell Line Generation

HEK293T cells (ATCC) were routinely maintained in Dulbecco's modified Eagle's medium supplemented with 10% fetal bovine serum (Gibco) and 100 *μ*g/ml noromycin. Transfection of HEK293T was performed using PEI as described previously [43]. Briefly, 10^5^ cells were seeded in 6-well plates one day before transfection, and 2 *μ*g of each plasmid was used for each well. To generate stable HEK293T cell lines harboring pHBRV Su-TAP-4C FW, the pHBRV Su-TAP-4C FW-puromycin plasmid was linearized with *Pvu*I and transfected into HEK293T cells. Individual clones were selected with puromycin (Invitrogen).

### 2.5. Western Blot Analysis

**Figure 1:**
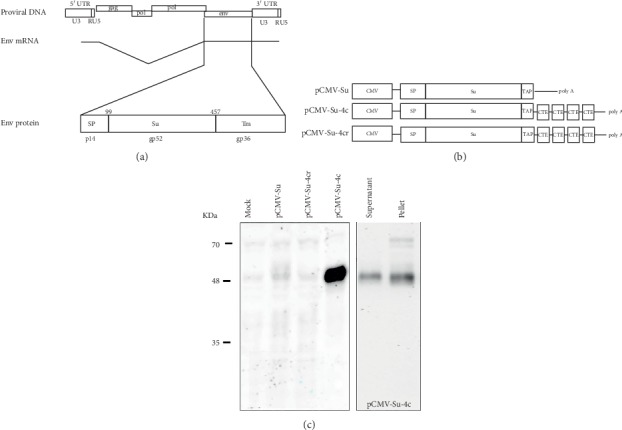
(a) The single spliced mRNA of betaretrovirus Env encodes the signal peptide, surface, and transmembrane proteins. (b) The HBRV Su construct used for mammalian expression contained the cytomegalovirus immediate early promoter, HBRV SP, and Su, a TAP tag; pCMV-Su-4c contained 4 copies of M-PMV CTE inserted in the downstream of Su-TAP in either the sense (pCMV-Su-4c) or the antisense (pCMV-Su-4cr) orientation. (c) Only the pCMV-Su-4c containing the CTE in the correct orientation produced sufficient HBRV Su protein in the cell pellet and supernatant as shown by the western blot analysis.

Secreted HBRV Su protein in 400 *μ*l supernatant was precipitated with TCA and dissolved in PBS. Cell lysates were prepared from transfected and stable cells using RIPA buffer with complete proteinase inhibitor (Roche). Approximately 2 × 10^6^ cells were collected and washed twice with ice-cold PBS, incubated with RIPA buffer on ice for 30 min, and centrifuged at 20,000 ×g for 30 minutes. Proteins from cell supernatant and lysate were quantified using the BCA assay (Bio-Rad), and 50 *μ*g and 100 *μ*g of total protein from cell lysate and supernatant, respectively, were resolved by 10% sodium dodecyl sulfate- (SDS-) polyacrylamide gel electrophoresis (PAGE) and transferred to nitrocellulose membrane as previously described (Figure 1) [44].

Western blot analysis was performed using the primary polyclonal goat anti-MMTV envelope gp52 antibody (kindly provided by Dr. Susan Ross, University of Pennsylvania), mouse monoclonal anti-Flag antibody (Sigma-Aldrich), and IRDye goat anti-mouse and rabbit anti-goat secondary antibodies (LI-COR). Reacting membranes were visualized with LI-COR Odyssey infrared imaging system. The anti-MMTV envelope gp52 antibody has demonstrable reactive biliary epithelial cells extracted from a liver transplant recipients with PBC (Supplementary Figure 2), previously shown to have HBRV infection with documented HBRV proviral integrations and HBRV RNA by the QuantiGene assay and *in situ* hybridization [30].

For detection of serological reactivity to HBRV Su, 100 ng of purified protein was resolved on a 10% SDS-PAGE minigel (Bio-Rad) and transferred to nitrocellulose membrane. The membrane was cut into 5 mm wide stripes. Each stripe was incubated with serum from a breast cancer patient or a control (1 : 400 dilution) and IRDye goat anti-human secondary antibody.

### 2.6. Scale-Up of HBRV Su Production and Purification and Characterization

Stable cells expressing HBRV Su were expanded to 12 × 15 cm cell cultural dishes in Dulbecco's modified Eagle's medium supplemented with 10% fetal bovine serum. The medium in each plate was replaced with 25 ml Pro293™ CD serum-free medium (Lonza) when cells reached 95% confluence. The medium was collected after 5-6 days of incubation and centrifuged at 3,000 g for 20 min. The clarified medium was adjusted to pH 8.0 and filtered through a 0.22 *μ*m filter before purification.

Purification of HBRV Su was performed on 1 ml Histrap FF crude column and buffers as suggested by the supplier (GE Healthcare) using an ÄKTA explorer 100 (Amersham Pharmacia Biotech). The conditioned medium was loaded to the equilibrated column at the rate of 1 ml/min, and the column was then washed with 20 ml binding buffer and eluted into 10 × 0.5 ml fractions using elution buffer. The peak elution fraction was combined and changed to proteins storage buffer by ultrafiltration (Millipore, 30 kDa cutoff limit concentrator, 4000 g for 20 min). The final preparation was aliquoted for storage at −80°C for ELISA. The 10 eluted fractions were assessed by western blot analysis using anti-MMTV Env antibody or anti-FLAG antibody and 10% SDS-PAGE gels stained with Coomassie R-250 blue stain (Bio-Rad). The protein concentration was determined by BCA assay (Pierce) using bovine serum albumin (BSA) as a standard.

### 2.7. HBRV Su ELISA

ELISA was performed at room temperature with all sera in duplicate using high-binding microplates (Greiner, Monroe, USA). Briefly, wells were coated with 100 *μ*l of 2 ng/*μ*l purified HBRV Su in PBS for 18 hours and blocked with 1% BSA in PBS for 3 hours. Serum was incubated at 100 *μ*l/well at a 1 : 400 dilution in PBS with 1% BSA (Sigma) for 1 hour. A serial dilution of polyclonal anti-MMTV Env was included on each plate as a standard and then incubated with 100 *μ*l/well donkey anti-human and donkey anti-goat secondary antibodies (Jackson Immuno-Research Lab) for 1 hour. The plate was washed 3 × 5 min after each step using PBS with 0.5% Tween. Plates were developed with 100 *μ*l/well tetramethylbenzidine substrate (TMB, Sigma) for 20 min and then stopped with 50 *μ*l/well 2N H_2_SO_4_. The absorbance at 450 nm and 540 nm (background) was measured with EMAX Plus Microplate Reader (Molecular Devices, USA) and the cutoff level was established using the reactivity of control samples by adding the mean background level to 3 × S.D. Two-tailed Fisher's exact test was used to assess significant differences in frequency between different groups, followed by calculation of the odds ratio (Baptista–Pike methodology) along with sensitivity, specificity, positive predictive value, negative predictive value, and likelihood ratio (Wilson Brown methodology) using Prism 8 software.

## 3. Results

### 3.1. HBRV Su Expression in HEK 293T Cells

A mammalian expression system was employed to express the HBRV Su because prior attempts to express multiple constructs expressing HBRV Env protein in bacteria and baculovirus systems were not sufficiently productive. MMTV Env protein is encoded by a single spliced mRNA in mice, which produces a signal peptide (SP p14), surface (Su gp52), and transmembrane domain (TM gp36) (Figure 1(a)); the Su protein is generated by removal of the signal peptide by signal peptidase and cleavage of the transmembrane domain by cellular Furin. Therefore, a mammalian expression vector pCMV Su-Tap was constructed, using the cytomegalovirus immediate early promoter to drive protein expression and a TAP tag to enable protein purification (Figure 1(b)). Using the pCMV-Su-TAP construct, very little HBRV Su protein was detected in lysates from transfected HEK293T cells (Figure 1(c)). Therefore, an M-PMV cytoplasmic transport element (CTE) was incorporated into the construct to increase protein expression [42]. To this end, two additional Su expression constructs were generated with the 4 copies of M-PMV CTE inserted in the downstream of Su-TAP for expression studies. Following expression in HEK293T, increased production of HBRV Su was observed in cell lysates transfected with the pCMV-Su-Tap-4c but not in cells with the pCMV-Su-Tap-4cr construct that had the CTE arranged in the antisense orientation. Moreover, we were able to detect secreted Su protein in the medium of the cells transfected with the pCMV-Su-Tap-4c plasmid two days after transfection (Figure 1(c)).

### 3.2. Large-Scale Production and Purification of HBRV Su

**Figure 2:**
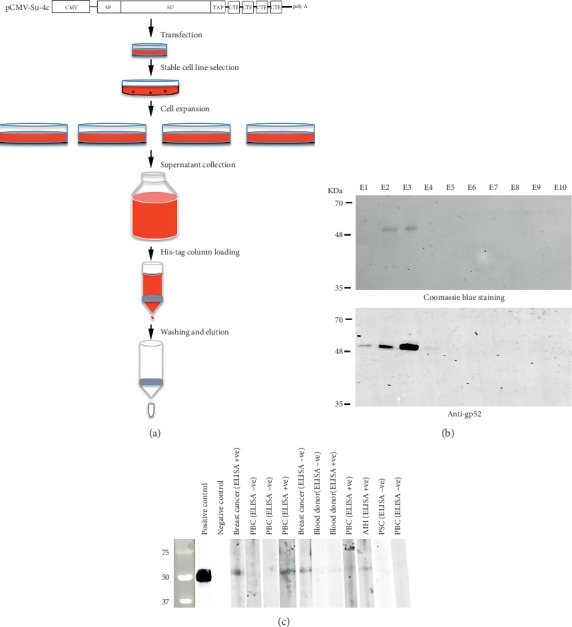
(a) Schematic showing large scale HBRV Su protein purification from the supernatant of HEK293T cells using a His-tag column. (b) Coomassie blue staining and western blot analysis demonstrate the purity of the HBRV gp52 protein using anti-MMTV gp52 Su in sequential elutions. (c) Western blot confirmation of ELISA positive and negative samples demonstrates reactivity using select breast cancer, PBC, and blood donor control samples. The breast cancer serum sample used in lane 7 is positive by western blot and negative by ELISA.

Since abundant HBRV Su protein was secreted from 293T cells transfected with the pCMV-Su-Tap-4c plasmids, a strategy was developed to purify the protein directly from a large-scale cell culture medium (Figure 2(a)). Stable 293T cell lines were generated following transfection with the pCMV-Su-Tap-4c plasmid and the cells with the highest Su secretion in the culture medium were expanded to 12 × 15 cm cell culture dishes using DMEM supplemented with 10% FBS. When cells reached 90–95% confluence, the medium was replaced with serum-free medium and incubated for another 5 days before collection. Approximately 300 ml was obtained for each batch, which was then purified with chromatography to derive 150–200 *μ*g HBRV Su protein. SDS-PAGE revealed that the purified Su protein was homogeneous and devoid of other contaminants. Western blot analysis with polyclonal anti-MMTV Env confirmed that the purified protein was HBRV Su along with select serum from seropositive and negative breast cancer and control samples (Figures 2(b) and 2(c)).

### 3.3. Detection of Anti-HBRV Su Protein Antibodies by ELISA

**Figure 3:**
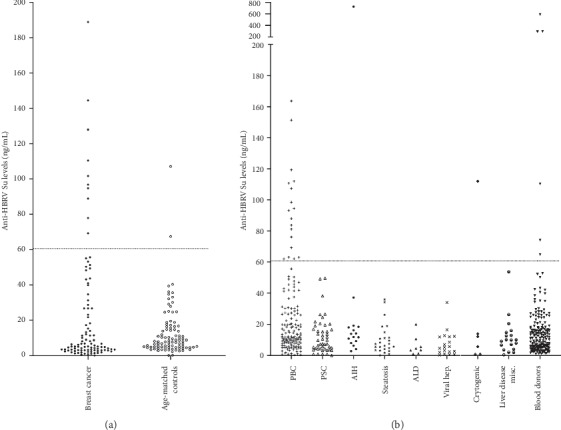
(a) A higher percentage of reactivity to HBRV Su was observed in breast cancer patients' sera versus age/sex-matched healthy controls (10/98 vs. 2/102; *P*=0.017). (b) Anti-HBRV reactivity was highest in patients with PBC (18/156) and found in AIH (1/16), cryptogenic liver disease (1/6), and healthy blood donors (6/194), whereas reactivity was not observed in patients with PSC, steatosis (NAFLD), ALD, or miscellaneous liver disease (PBC vs. blood donors 11.5% vs. 3.1%, *P*=0.0024, OR = 4.09 [1.66–10.1]).

The ELISA protocol was established using 200 ng/well of purified HBRV Su. The antibody response was calculated by converting the optical density reading to the equivalent  ng/ml reactivity of the positive control, polyclonal anti-MMTV Env antibody. The background reactivity was calibrated using the serum samples from the age/sex-matched healthy controls used as a comparison group for the breast cancer patients. The cutoff level (mean background + 3 × S.D.) was calculated as 61 ng/mL and samples found to be greater than this were considered positive (Figure 3). Accordingly, a greater proportion of breast cancer patients (10.2%) were found to have serological reactivity to HBRV Su versus 2.0% of age- and sex-matched control subjects (Figure 3: *P*=0.017, OR = 5.6 [1.25–26.3]).

The seroprevalence of HBRV Su reactivity in patients with PBC was comparable to that observed in patients with breast cancer (Figure 3: 11.5% vs. 10.2%). The frequency of HBRV Su reactivity was significantly higher in PBC patients vs. blood donors (11.5% vs. 3.1%, *P*=0.0024, OR = 4.09 [1.66–10.1]). In prior studies using the gold standard methodology of detecting HBRV integrations in patients' cholangiocytes, subjects with cryptogenic liver disease and AIH were found to harbor infection, and in this study, isolated reactivity was observed in subjects with cryptogenic liver disease (16.7%) and AIH (6.3%), whereas other subjects with liver disease were universally negative (Figure 3(b)).

While reactivity in healthy blood donors was incrementally higher than the healthy age/sex-matched comparison group for the breast cancer patients, the difference was not found to be significant (3.1% vs. 2.0%; *P*=0.72). The sensitivity of the HBRV Su ELISA was limited in detecting reactivity in patients with breast cancer and PBC as compared to their respective control groups (10.2% [5.6%–17.8%] and 11.5% [7.4%–17.5%]), whereas the assay was highly specific for serologic detection in patients with breast cancer and PBC, respectively (98.0% [93.1%–99.7%] and 97.0% [93.4%–98.6%]). Accordingly, the positive predictive values (83.3% [55.2%–97.0%] and 75.0% [55.1%–88.0%]) were diagnostically more useful that the negative predictive values (53.2% [46.1%–60.2%] and 57.7% [52.3%–62.9%]) for patients in the breast cancer and the liver disease study groups.

## 4. Discussion

This is the first report using an HBRV ELISA for assessing the seroprevalence of infection in patients. Approximately 10% of breast cancer and PBC patients had detectable anti-HBRV Su, and the test was found to be highly specific for both disorders. The likelihood ratio for having breast cancer with HBRV Su reactivity was 5.2 and for having PBC with HBRV Su reactivity was 3.7; the difference in likelihood ratios probably reflects the chosen control groups for each disorder. Notably, the breast cancer control subjects were mainly middle-aged women and therefore a more suitable control group for the PBC patients, who are also predominantly female; whereas the blood donors were more of an admixture of both sexes. The healthy comparison groups revealed a sizeable population seroprevalence of ∼2-3%. These data are in keeping with the hypotheses that HBRV infection may only be disease related in genetically predisposed individuals [10, 45].

Prior seroprevalence studies using MMTV proteins have been widely inconsistent. For example, an ELISA-based study using MMTV proteins demonstrated serological reactivity in 26% of breast cancer patients and 8% of healthy controls [46]. A similar study found no difference between breast cancer patients and their respective controls [47] and a study using 4 strains of MMTV reported only nonspecific reactivity in breast cancer patients, although reactivity consistent with the molecular weights of viral proteins was observed in individual strains of MMTV [39]. In studies of patients with liver disease, MMTV western blot reactivity was attributed to autoreactivity with the antimitochondrial antibody, which is found in up to 95% of patients and used for diagnosing PBC [29], whereas similar MMTV western blot studies employing mitochondrial proteins to remove the autoantibodies from PBC patients' serum demonstrated the presence of signal to the betaretrovirus gp52 surface protein [48]. As the purified antimitochondrial antibody has no reactivity with HBRV Su, we can conclude that humans do make humoral responses to HBRV based on our ELISA.

A second issue to be addressed is that the prevalence of infection detected by the HBRV Su ELISA was somewhat lower than other reports using different techniques to diagnose disease. Indeed, our western blots (Figure 2(c)) show reactivity to one breast cancer sample that was negative by the ELISA, suggesting that our cutoff level may have been too stringent. Using nonserological techniques, a meta-analysis of molecular epidemiological studies reported a prevalence of 40% HBRV infection in Western countries based on PCR detection of betaretrovirus sequences in breast cancer samples [49]. An even higher prevalence of infection has been reported in PBC patients based on the presence of proviral HBRV integrations detected by ligation-mediated PCR and Illumina sequencing, with provirus found in 58% of cholangiocytes from patients with PBC as compared to 7% of nonautoimmune liver disease controls [30]. The discrepancy of a higher frequency of viral infection in tissue as compared to a lower seroprevalence of anti-HBRV Su reactivity may be partly explained by observations from neonatal mouse infection. Weanling pups have a high risk of developing breast cancer from MMTV infection because they become immunotolerant to viral infection. This occurs because MMTV is taken up in the gut-associated lymphoid tissue along with bacterial lipopolysaccharide, which triggers a cascade of events. The lipopolysaccharide/viral complex engages Toll-like receptor 4 that in turn triggers an IL-4- and IL-6-dependent production of IL-10, which renders the mouse unresponsive to MMTV Su and prevents the formation of neutralizing antibodies [13]. It is currently unknown whether a similar immunological process may occur in humans with HBRV infection. Notably, the cellular immune response to HBRV peptides is more prevalent in patients with liver disease [50].

Our overall goal was to derive a reliable and reproducible diagnostic ELISA to investigate the frequency of HBRV infection. In prior experiments, we used bacterial or baculovirus expressed proteins but failed to generate sufficient amounts of pure viral protein. We also generated serological data using the bacterially expressed Gag proteins, and while a higher seroprevalence was observed in our PBC population as a whole, no significant differences were found between patients and controls with liver disease. Notably, cross reactivity with retroviral Gag (Group AntiGen) is a common occurrence in patients with any viral infection due to the positively charged antigenic determinants in capsid and core proteins surrounding the viral genome [51, 52]. For this ELISA, a novel strategy for large-scale production of purified and secreted HBRV Su protein was developed using HEK 293T cells. Three factors contributed to the production of HBRV Su sufficient for multiple ELISAs: these included (i) using multiple copies of CTE downstream of the Su coding region to enhance HBRV Su expression and secretion; (ii) ensuring the stable expression of HBRV Su protein in human cells; and (iii) replacing the FBS containing medium with serum-free medium to remove a source of protein contamination and ensure the high purity of protein after chromatography purification. We can also speculate that the use of HBRV rather than MMTV proteins to assess the betaretrovirus seroprevalence likely improved the accuracy of the assay. Nevertheless, more sensitive assays employing cellular immune responses to viral peptides [50], for example, will be required to improve the sensitivity for the detection of immune response to HBRV.

## 5. Conclusions

An HBRV ELISA has been constructed by expressing *HBRV env* in HEK293 to produce purified HBRV Su protein. The ELISA detection of HBRV Su antibodies is highly specific for both breast cancer and PBC, but the assay may lack sensitivity as higher prevalence rates for HBRV infection have been recorded using other techniques. Further studies may permit testing whether anti-HBRV is linked with breast cancer, by screening archived predisease serum from patients participating in the Alberta Tomorrow Project who subsequently developed breast cancer. Accordingly, we will be able to study whether anti-HBRV Su predates the development of disease and may act as a biomarker for breast cancer.

## Data Availability

The data used to support the findings of this study are included within the supplementary information files and available on request from the corresponding author.

## Supplementary Materials

Supplementary Materials HBRV Su expression construct and coding sequences; alignment of HBRV Su and MMTV Su proteins as well as anti-MMTV gp52 Su reactivity to biliary epithelial cells cultured from a PBC patient's resected liver following liver transplantation.
