# Corrigendum to “Seroprevalence of Human Betaretrovirus Surface Protein Antibodies in Patients with Breast Cancer and Liver Disease”

**DOI:** 10.1155/2020/7406146

**Published:** 2020-12-03

**Authors:** Guangzhi Zhang, Kiandokht Bashiri, Mark Kneteman, Kevan Cave, Youngkee Hong, John R. Mackey, Harvey J. Alter, Andrew L. Mason

**Affiliations:** ^1^Center of Excellence for Gastrointestinal Inflammation and Immunity Research, Division of Gastroenterology, University of Alberta, Edmonton AB T6G 2E1, Canada; ^2^National Microbiology Laboratory, Winnipeg MB R3E 3M4, Canada; ^3^Department of Medical Oncology, Cross Cancer Institute, University of Alberta, Edmonton, AB, Canada; ^4^Department of Transfusion Medicine, National Institutes of Health, Bethesda, MD 20892, USA; ^5^Li Ka Shing Institute of Virology, University of Alberta, Edmonton AB T6G 2E1, Canada

In the article titled “Seroprevalence of Human Betaretrovirus Surface Protein Antibodies in Patients with Breast Cancer and Liver Disease” [1], a sentence in Section 2.2 was incorrect. The sentence “A serum panel of breast cancer patients (*n* = 98) and age/sex-matched controls (*n* = 102) was obtained from the Alberta Tomorrow Project, a longitudinal study tracking 55,000 adults in Alberta” should be corrected to “A serum panel of breast cancer patients (*n* = 98) and age/sex-matched controls (*n* = 102) was obtained from the breast cancer outpatient clinic Cross Cancer Institute.” The authors also wish to acknowledge an additional funding body. The modified Acknowledgments section is as follows.
