# Supplementary material for: Seroprevalence of Human Betaretrovirus Surface Protein Antibodies in Patients with Breast Cancer and Liver Disease
Source: J Oncol. 2020 Jan 27;2020:8958192. doi: 10.1155/2020/8958192 (PMC7204138; doi:10.1155/2020/8958192)

**Supplementary Material**

**Seroprevalence of Human Betaretrovirus Surface Protein Antibodies in Patients with Breast Cancer and Liver Disease**

Guangzhi Zhang, Kiandokht Bashiri, Mark Kneteman, Kevan Cave, Youngkee Hong, John R. Mackay, Harvey J. Alter, Andrew L. Mason

**Supplementary Material:**

**HBRV Su expression construct**

The pHBRV Su-TAP expression construct was made with the TAP tag located at the 3’ terminus of the HBRV Su. The TAP, HBRV Signal peptide and Su coding region (1362 bp) were PCR-amplified with primer pairs SuFW/SuRV and TapFW/TapRV (Table) using HiF Taq polymerase (Invitrogen). The amplified fragments were digested with *NheI/KpnI* and *KpnI/ BamHI* and cloned into pcdna3.1 (Invitrogen). To generate pHBRV-SU-TAP-4CFW and pHBRV-SU-TAP-4CRV, and the CTE sequence was excised from pGAG-GFP-CET with Xbal and inserted into the corresponding site of pHBRV Su-TAP. To create pHBRV Su-TAP-4C FW-puromycin, the puromycin open reading frame was amplified from pCMV-MMTV-puri [Zhang, G., et al., *Pericentriolar Targeting of the Mouse Mammary Tumor Virus GAG Protein.* PLoS One, 2015. **10**(6): p. e0131515] with primer pair puriFW/puriRV and inserted into the *Sma*l/*Bst*BI sites of pHBRV Su-TAP-4C. All the constructs were verified by sequencing.

**Table.** Primer sequences used for construction of the expression plasmids

| Primer | Primer sequence (5’ to 3’) ^*^ | Restriction enzyme |
| --- | --- | --- |
| SuFW | GTTGGCTAGCATGCCGAATCACCAATCTGGGTCC | *NheI* |
| SuRV | TCGAGGTACCGGCTCGAATTAAATCTGTGGCAT | *KpnI* |
| TapFW | ATGCGGTACCCTGGTGCCGCGCGGCAGCG | *KpnI* |
| TapRV | CTCCGGATCCTTAATGGTGATGGTGATGATGCC | *BamHI* |
| PuriFW | GATCGATATCCCGGGATGGCCACCGAGTACAAGCCCAC | *Smal* |
| PuriRV | GATCTTCGAATCAGGCACCGGGCTTGCGGGTC | *BstB1* |

* Introduced restriction sites are underlined.

**HBRV envelope coding sequence [1362 bp, primers underlined]**

The HBRV Su was constructed from Human betaretrovirus isolates AF513920, AF513921 and AY326252 [Xu, L., et al., *Cloning the human betaretrovirus proviral genome from patients with primary biliary cirrhosis.* Hepatology, 2004. **39**(1): p. 151-6].

atgccgaatcaccaatctgggtccccgaccggttcatccgaccttttactgagcggaaagaagcaacgcccacacctggcactgcggagaaaacgccgcagcgagatgagaaagatcaacaggaaagtccggaggatgaatctagcccccatcaaagagaagacggcttggcaacatctgcaggcgttaatcttcgaagcggaggaggttcttaaaacctcacaaactccccaaacctctttgactttatttcttgctttgttgtctgtcctcggccccccgcctgtgaccggggaaagttattgggcttacctacctaaaccacctattctccatcccgtgggatggggaaatacagaccccattagagttctgaccaatcaaaccatatatttgggtgggtcacctgactttcacgggtttagaaacatgtctggcaatgtacattttgaggggaagtctgatacgctccccatttgcttttccttctccttttctacccccacaggctgctttcaagtagataagcaagtatttctttctgatacacccgcggttgataataataaacctgggggaaagggtgataaaaggcgtatgtgggaactttggttgactactttggggaactcaggggccaatacaaaactggtccctataaaaaagaagttgccccccaaatatcctcactgccagatcgcctttaagaaggacgccttctgggagggagacgagtctgctcctccacggtggttgccttgcgccttccctgaccagggggtgagtttttctccaaaagggacccttgggttactttgggatttctcccttccctcgcctagtgtagatcagtcagatcagattagaagcaaaaaggatctatttggaaattatactccccctgtcaataaagaggttcatcgatggtatgaagcaggatgggtagaacgtacatggttctgggaaaattctcctaaggatcccaatgatagagattttactgctctagttccccatacagaattgtttcgcttagttgcagcctcaagatatcttattctcaaaaggccaggatttcaagaacatgacatgattcctacatctgcctgtgctacttacccttatgccatattattaggattacctcagctaatagatatagagaaaagaggatctacttttcatatttcctgttcttcttgtagattgactaattgtttagattcttctgcctacgactatgcagcgatcatagtcaagaggccgccatacgtgctgctacctgtagatattggtgatgaaccatggtttgatgattctgccattctaacctttaggtatgccacagatttaattcgagcc

**Supplemental Figures**

**Sup Fig. 1 HBRV and MMTV Alignment**

Alignment of expressed HBRV Su amino acid sequence 99 to 455 showing 97%-98% identity with MMTV derived from the BR6 (MMTVB Su), C3H (MMTVC Su) and the GR strain (MMTVG Su). Four consistent differences in the HBRV Su compared to MMTV Su are illustrated here with the variants at **S**11**P**, **L**65**F**, **T**171**A,** and **L**339**F.** Whereas the variant **D**265**E** seen below is not observed in other HBRV Su clones (AAP73835, ref 6) and the **I**283**L** variant is observed in MMTV AAF31475; therefore neither of these variants are specifically associated with HBRV vs MMTV.


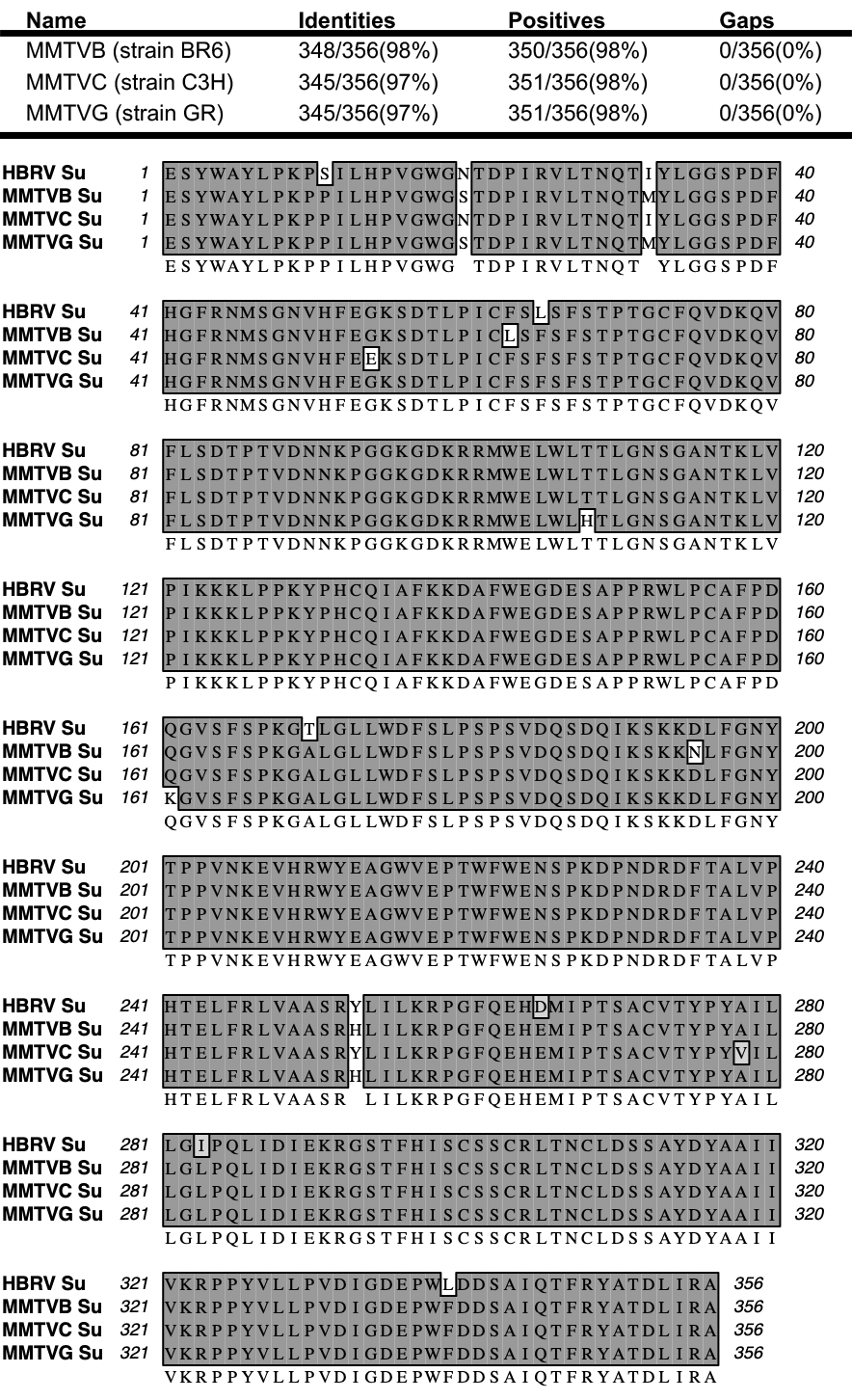


**Sup Fig. 2 Anti-MMTV gp52 Su immunocytochemistry**

(A) Polyclonal anti-MMTV gp52 Su shows no reactivity to control human biliary epithelial cells extracted from liver transplant recipients. (B) Cell surface reactivity is observed on PBC biliary epithelial cells using anti-MMTV gp52 Su for immunocytochemistry studies. Prior studies have reported HBRV infection in the majority of PBC biliary epithelium by demonstration of HBRV integration, and/or HBRV RNA by *in situ* hybridization and QG assay (ref 30).


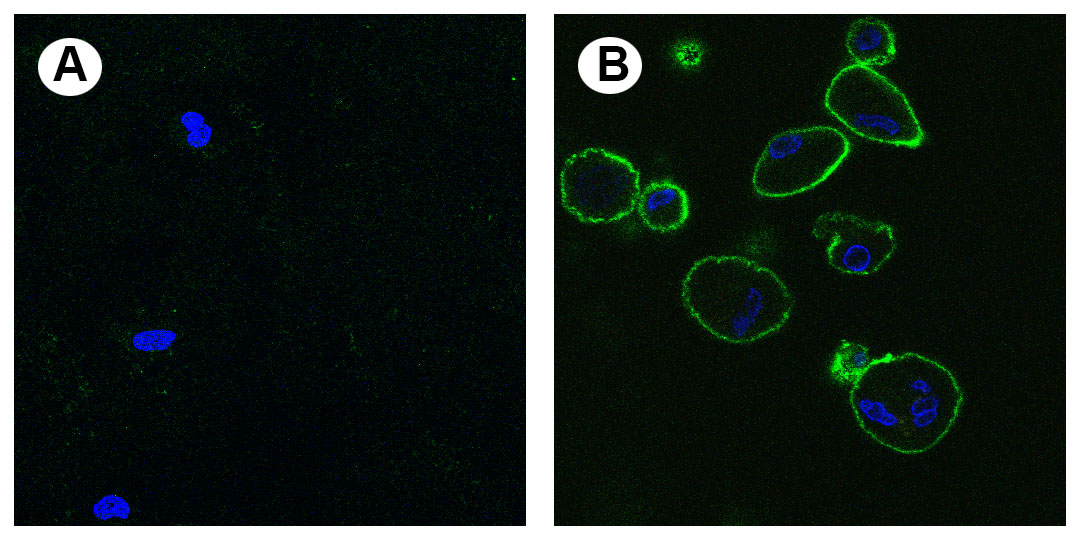

Supplement: Supplementary Materials — HBRV Su expression construct and coding sequences; alignment of HBRV Su and MMTV Su proteins as well as anti-MMTV gp52 Su reactivity to biliary epithelial cells cultured from a PBC patient's resected liver following liver transplantation. [file 8958192.f1.docx]
